# miR-371b-5p-Engineered Exosomes Enhances Tumor Inhibitory Effect

**DOI:** 10.3389/fcell.2021.750171

**Published:** 2021-10-04

**Authors:** Qiang Xue, Yang Yang, Linlin Yang, Xiaodi Yan, Zihao Shen, Jiajia Liu, Jianhua Xue, Wei Zhao, Xianchen Liu

**Affiliations:** ^1^Department of Radiation Oncology, Affiliated Hospital of Nantong University, Nantong, China; ^2^Department of Trauma Center, Affiliated Hospital of Nantong University, Nantong, China; ^3^Department of Oncology, Sheyang People’s Hospital, Yancheng, China; ^4^Medical College, Nantong University, Nantong, China; ^5^Department of Biomedical Sciences, City University of Hong Kong, Kowloon, Hong Kong, SAR China

**Keywords:** engineered exosomes, miR-317b-5p, tumor cells, proliferation, migration, xenograft

## Abstract

**Background:** Exosomes are well-known natural nanovesicles, that represent one of the recently discovered modes of intercellular communication due to their ability to transmit cellular components. Exosomes have been reported to have potential as natural vectors for carrying functional small RNAs and delivering chemotherapeutic agents to diseased cells. In this study, we aimed to investigate the role of exosomes in carrying miRNA for targeting tumor cells.

**Methods:** We present a novel method for engineering exosomes with functional miR-317b-5b to target tumor cells. MiR-317b-5b exerts its anti-tumor function *via* its expression in tumors. RT-qPCR was performed to assess the levels of miR-371b-5p, FUT-4. Western blot was performed to measure the levels of CD9, CD81, and FUT-4 proteins. Confocal microscopy was used to observe the internalization of miR-317b-5b in tumor cells. CCK-8, EdU, flow cytometry, wound-healing migration and transwell assays were performed to evaluate cell viability, proliferation, migration, and invasion, respectively.

**Results:** Our findings illustrated that miR-317b-5b-loaded engineered exosomes were internalized by tumor cells. MiR-317b-5b was overexpressed in tumor cells treated with miR-317b-5b-loaded engineered exosomes. The internalization of miR-317b-5b in tumor cells was accompanied by changes of cell viability, proliferation, apoptosis, and migratory and invasive capability. We found that miR-317b-5b-loaded engineered exosomes were presence in tumor tissue sections and miR-317b-5b was overexpressed in tumor tissues of osteosarcoma tumor-bearing mice infected with miR-317b-5b-loaded engineered exosomes. MiR-317b-5b-loaded engineered exosomes had the anti-tumor efficiency *in vivo*.

**Conclusion:** Our findings show that miR-317b-5b-loaded engineered exosomes can be used as nanocarriers to deliver drug molecules such as miR-317b-5b both *in vitro* and *in vivo* to exert its anti-tumor functions.

## Introduction

Extracellular vesicles (EVs), that contain exosomes, microbubbles and apoptotic bodies are produced by various cell types including mesenchymal stem cells, endothelial, epithelial, and tumor cells (Sutaria et al., 2017). Exosomes contain various macromolecular substances with the size of approximately 30–150 nm, and these macromolecules mediate local or systemic intercellular communications (El Baradie et al., 2020).

In recent years, studies on exosomes have developed into many practical applications, including biomarkers of diseases and therapeutic responses, and drug carriers (Marcus and Leonard, 2013; Hong et al., 2014; Batrakova and Kim, 2015). Different from synthetic liposomes or nanoparticles, exosomes composed of natural ingredients that could avoid clearance in blood circulation (Lundqvist et al., 2008; Kopac, 2021). The exosomes transport their cargos to recipient cells by adhesion proteins or direct fusion with the plasma membrane. At present, exosomes are explored to be used as drug carriers for siRNA, miRNA simulators, miRNA inhibitors, mRNAs, and proteins expressed by plasmid DNAs (Mulcahy et al., 2014).

The main challenge of gene therapy is to develop non-toxic molecular transporters, that can effectively deliver functional copies of therapeutic genes to target cells (Niidome and Huang, 2002). Exosomes can transport mRNA, miRNAs, and proteins to remote target cells *via* the specific interaction between proteins on exosome membrane and receptor molecules on target cells (Fuhrmann et al., 2015). Hence, the construction of engineered exosomes has become one of the research hotspots of gene therapy. Previous studies have shown that the molecular cargo delivered by engineered exosomes to target cells can affect the pathophysiological processes of target cells or tissues, including cell biological activity, tumor growth, and tissue repair (Gidlöf et al., 2013; Tian T. et al., 2014; Qiu et al., 2018). In addition, the presence of specific genetic information in exosomes derived from tumor cells provides an opportunity for cancer detection or monitoring the effectiveness of cancer therapy (Ghai and Wang, 2016).

Studies have shown that most miRNAs are differentially expressed during tumorigenesis (Calin and Croce, 2006). MiRNAs are promising therapeutic targets because they could regulate an entire signaling pathway rather than a single protein. If they are overexpressed in tumors, they are easily inhibited by anti-miRNAs. If they are under expressed, they may be supplemented by miRNA mimics (Henry et al., 2010). Furthermore, MiRNAs can be used as molecular therapy for a variety of diseases, particularly cancers (O’Connor et al., 2016). Most therapeutic applications of miRNA require packaging nucleic acids in vectors or nanocarriers. At present, the packaging of miRNAs into exosomes has been widely studied (Rincon et al., 2015; Nie et al., 2020). It was known that MiR-317b-5p is involved in tumor cell proliferation, migration, and invasion and is differentially expressed in various cancers, including non-small cell lung cancer, chondrosarcoma, and bladder cancer (Mutlu et al., 2015; Li et al., 2020; Luo et al., 2020).

In this study, miR-317b-5b-loaded engineered exosomes were constructed to explore the effect of miR-371b-5p in tumor pathological behaviors. We developed an active delivery method that uses the interaction of HIV-1 TAR RNA-TAT peptides to exchange pre-miR-317b-5p rings with TAR RNA rings. The pre-miR-317b-5p, modified with LAMP2A fusion protein, and identifies TAT peptides in electric vesicles. Using this TAT-TAR interaction, the load of miR-317b-5p to the outer cut can be enhanced (Sutaria et al., 2017; Liang et al., 2018).

## Materials and Methods

### Mice

Female BALB/c mice, 4 to 8 weeks old, purchased from Shanghai Sippr-BK Experimental Animals Co., Ltd., (Shanghai, China). The mice were kept in cages in airy rooms with free access to food and water. The animal experiments are conducted in accordance with national-specific ethical standards for biomedical research and approved by the animal ethics committees of Nantong University.

### Cell Culture

The 143B, HeLa, and A549 tumor cells were provided by Procell Life Science and Technology Co., Ltd., (Wuhan, China). The cells were cultured in Minimum Essential Medium (MEM, Roche, Basel, Switzerland) containing 1% penicillin Streptomycin Solution and 10% FBS (Solarbio, Beijing, China). All cells were cultured in a 37°C humidified incubator containing 5% CO_2_.

### Construction of Plasmids

Supplementary Figure 1 summarizes the process by which miR-317b-5p is incorporated into the HEK293T exosome. The pEGFP-1 carrier expressing Lamp2b is provided by Procell Life Technology Co., Ltd., (Wuhan, China). The LAMP2A was cloned into the carrier skeleton using *Nhe*I and *Bam*HI enzyme inching points. Four peptide sequences were introduced into the LAMP2A sequence: 3X Flag peptides for exosome purification, PC94 peptides for liver cancer targeting, TAT peptides combined with modified TAR, and His tag for verifying the protein is translated within the framework. Because HEK293T cells are resistant to neomycin, a methromycin box is introduced downstream of the SV40 initiator. Human miR-317b-5p was inserted into the artificial intron of LAMP2A gene. The miR-317b-5p sequence was cloned into the exon binding site of LAMP2A gene.

### Preparation of Exosome

According to the previous research, the engineering exosomes was separated by overspeed centrifugal method (Théry et al., 2006). About 360 mL 293T cell culture (approximately 4 × 10^8^ cells) was centrifuged at 300 *g* for 10 min, moved into a clean test tube and centrifuged again at 2,000 *g* for 20 min. The cell fragments were removed. Subsequently, the fluid was transferred into a clean test tube, centrifuged at 10000 *g* for 30 min, and filtered in a vacuum with a 0.22 M filter (EMD, Billica, MA, United States). Furthermore, the supernatant was transferred into a high-speed centrifuge tube (Beckman Coulter, Braille, CA, United States) and centrifuged at 11,000 *g* for 4 h in the overspeed centrifuge (Beckman Coulter Optima TM Xe, Billica, MA, United States). The sediment was rinsed once with sterile phosphate buffer (PBS, pH 7.4) and then suspended in a 0.3 to 0.6 mL PBS containing 1% DMSO. The outer cut^TM^ was prepared with APILES. The protein contents of exosomes were measured using the Pierce^TM^ BCA protein kit.

### Characterization of Exosomes

The images of exosomes were taken with a transmission electron microscope (TEM). Briefly, the exosome sediment was washed with PBS three times, 10 min each time, and then fixed in PBS containing 2.5% dialdehyde at 4°C. Subsequently, exosomes were dehydrated in increasing concentration of alcohol. The samples were observed under 80kV TEM (JEM-2100; JEOL, Tokyo, Japan). The particle size of exosomes was measured using Zetasizer Nano S (Malvern Instrument, Malvern, United Kingdom). The surface charge was evaluated by measuring the Zeta level in the PBS buffer.

### Exosome Labeling and Loading

The exosomes were labeled with the fluorescent probe PKH26 (Invitrogen, Carlsbad, California, United States). The purified exosomes were incubated with 5 μL PKH26 at 37°C for 15 min, and the free probe was removed by centrifugation at 120,000 *g* for 90 min. After two washing and centrifugation cycles, the labeled exosomes were suspended in PBS and used for cell studies.

The exosomes with a total protein concentration of 10 μg/ml were mixed with the 400 nm Cy5 labeled miR-317b-5p in a 1 mL phosphate buffer. The mixture was electrophoresis at 400V, 50 sf, 30 ms pulse/2 s, and suspended for three cycles. After loading miR-317b-5p, it was diluted 10 times with PBS and centrifuged at 110,000× *g* for 70 min. The free miR-317b-5p was removed. RT-PCR was used to detect miR-317b-5p in the exosomes.

### Real-Time Quantitative Polymerase Chain Reaction (RT-qPCR)

The total RNA was extracted with TRIZOL reagent (Takara, Kusatsu, Japan), and the concentration of RNA was measured at 260 nm. M-MLV reverse transcriptase (RNase H) kit (Takara, Kusatsu, Japan) was used to synthesize cDNA. RT-qPCR was performed by using SYBR Green PCR Master Mix (Takara, Kusatsu, Japan). GAPDH was used as a negative control for mRNA detection, and U6 was used as a negative control for miRNA detection. The reaction was conducted under the following condition: template denaturation at 95°C for 3 min, followed by 40 cycles of denaturation at 95°C for 15 s, annealing at 60°C for 30 s, and extension at 60°C for 15 s. The 2^–ΔΔCT^ method was used to calculate the relative expression. The primer applied to this study were shown in Table 1.

**TABLE 1:** Primer sequences.

Primer name	(5′-3′)Primer sequences
F-miR-371b-5p	5′-GTGGCACTCAAACTGT-3′
R-miR-371b-5p	5′-CATCTTTTGAGTGTTAC−3′
F-FUT-4	5′-CAGTGGCCCGCTACAAGTTC-3′
R-FUT-4	5′-GCCAGAGCTTCTCGGTGATATAA−3′
F-U6	5′-CTCGCTTCGGCAGCACA−3′
R-U6	5′- AACGCTTCACGAATTTGCGT−3′
F-GAPDH	5′-GAGTCAACGGATTTGGTCGT−3′
R-GAPDH	5′-TTGATTTTGGAGGGATCTCG−3′

### Exosome Uptake

HeLa, 143B, and A549 cells (3 × 10^5^) were seeded in a 3.5 cm glass-bottom culture dish and cultured to 70% confluence. Subsequently, cells were washed with PBS and incubated with cell culture medium containing 10^8^ particles/mL of exosomes labeled with PKH26 Laser scanning confocal microscope (CLSM) was used to scan fluorescence images to record the fluorescent signals of PKH26 and to process images using the Zen software (CLSM; Zeiss lsm710, Oberkochen, Germany).

### Western Blot

Total proteins were separated with cell lysis buffer (Beyotime, Nanjing). The protein was analyzed with a 10% decanyl sulfate polyacrylamide gel (SDS-PAGE) and transferred to a TBST sealed polyfluoroethylene (PVDF) film in 5% skimmed milk powder, which was 1 h warmed at room temperature. Anti-FUT-4 antibody (Ab188610), anti-calcitonin antibody (Ab76011), anti-CD81 antibody (Ab79559), and anti-CD9 antibody (Ab92726) were incubated at 4°C overnight. β-actin (AB8226) was used as the internal control. Subsequently, membranes were incubated 2 h at room temperature with a secondary antibody (1:2,000). All antibodies were purchased from Abcam (Cambridge, United Kingdom, 1:1000). The optical density of protein band was determined by ImageJ software, Inc.

### CCK-8 Assay

Cell Counting Kit-8 (Beyotime, Nanjing, China) was used to assess the viability of cells. Briefly, the cells were inoculated to 96 well plates (3650, Corning, NY, United States) for 2 h. Subsequently, 10 μL of CCK-8 solution was added to the cell wells, incubated at 37°C for 2 h, and finally, the fluorescent microplate was used to detect the light absorbance reflecting the cell viability at 450 nm.

### EdU (5-Ethynyl-2’-deoxyuridine) Assay

After inoculation of 143B cells treated with engineered exosomes on a 24-well plate for 24 h, the EdU media was added. After incubation for 2 h, the culture medium was removed, the cells were digested with trypsin, and then washed twice with 1 × PBS. Cells were fixed with formaldehyde 30 min, decolored with glycine, and washed in PBS for two times. Subsequently, cells were soaked in 0.5% Triton X-100 for 10 min, and then washed twice with 1 × PBS. Finally, the staining was performed using the Cell Light^TM^ EdU Cell Proliferation Assay (Sigma, St. Louis, MO, United States) according to a previously published report (Xiao et al., 2019).

### Wound-Healing Migration Assay

The effect of miR-317b-5b engineered exosomes on the migration of 143B cells was quantitatively tested *in vitro*: 3 × 10^5^ 143B cells were inoculated into six well plates and incubated to reach 70% confluence (about 24 h). These cells were treated with engineered exosomes loaded with miR-317b-5b and incubated at 37°C for 4 h. A scratch wound was created using a sterile pipette tip. The cells were incubated in fresh culture medium at 37°C, and images of scraped monolayer cells were recorded at 0 and 48 h.

### Transwell Assay

Cell migration was evaluated in transwell chambers with a membrane pore size of 8 μm. 143B cells treated with engineered exosomes were inoculated in the top well of Millicell suspension culture plate (PIEP12R48) in triplicate with a density of 1 × 10^5^. The chamber insert was placed in a 24-well transplate containing DMEM and 30% fetal bovine serum as a chemical inducer. After 24 h incubation, the cells in the upper chamber were taken out with a cotton swab, and the cells on the lower surface of the membrane were fixed with 100% methanol for 15 min and stained with 0.05% crystal violet. Cells were counted at five random sites in each chamber and the mean number of cells was determined.

### Flow Cytometry Assay

Fluorescence Activated Cell Sorter (FACs) were performed to evaluate cell apoptosis. In brief, cells were inoculated to 96-well plates (3650; Corning, NY, United States) for 2 h. Heterocyanate fluorin (FITC) and propylene iodide (PI) were added to A549, HeLa, and 143B cells (5 μL/well) and incubated at 37°C for 2 h. The number of apoptotic cells was counted by flow cytometry. Apoptosis is defined as FITC (+) and PI (+).

### *In vivo* Visualization of Intravenously Injected Exosomes

In order to evaluate the biological distribution of miR-317b-5b containing exosomes, female BALB/c mice between 6 and 8 weeks of age were received subcutaneous flank injections of 1 × 10^6^ 143B cells tumor cells. Mice with tumors were monitored daily. When the tumor volume reaches 400 mm^3^, the 30 mg PKH26 labeled exosomes were injected into the nude mouse and the tumor-free mouse was used as a control. The 200 μL PBS + 5% glucose was injected into the abdominal cavity of the lotus mouse as a background control. Tumor bearing mice were anesthetized with 2.5% isoflurane at 3, 12, 24, and 48 h after administration, and small animal imaging system (Kodak, Rochester, NY, United States) was used. The fluorescent signals of each tissue sample were quantified in the target area drawn with the free hand.

### *In vivo* Anti-tumor Analyses

The 143B cells were injected subcutaneously into the ventral side of female BALB/c mice, and 1 × 10^6^ cells in 100 μL PBS were used. On the 12th day after inoculation, when the tumor was touchable, the mice were divided into four different treatment groups. The treatment group includes PBS, Unload Exo, NC Exo, and miR-317b-5b-Exo (30 μg, iv each). The tumor growth rate was measured with a caliper every 4 days. The survival of mice was monitored for 40 days. The probability of survival in mice was assessed by Kaplan–Meier method, and the survival ratio of each group was compared with the number of rank tests. The mice were sacrificed by cervical dislocation and the tumor was removed for further study.

### Tissue Dissection and Fluorescent Microscopy

The tumor tissue and the important organs (lung, liver, kidney, and spleen) were removed, immersed in 5, 10, and 14% sucrose, frozen with an OCT culture base at −80°C, and sliced with a Leica cm1800 cryostat. Frozen tissue slices (8 m thick) were fixed with acetone and the nuclei were dyed with DAPI. Fluorescently labeled exosomes were displayed using the Circle-3 Cell Imaging Multi-Mode Reader (Bio-Tek Instruments, Winooski, VT, United States).

### Statistical Analysis

Data were presented as mean ± standard deviation (SD) from three independent experiments. GraphPad Prism 5.0 Software (GraphPad Software, Inc.) was used for statistical analysis. *T*-test or one-way analysis of variance was used for comparison between two or more groups, and Tukey post-test was used for comparison within multiple groups. When *P* < 0.05, the difference was statistically significant.

## Results

### Isolation and Identification of Engineered Exosomes

**FIGURE 1:**
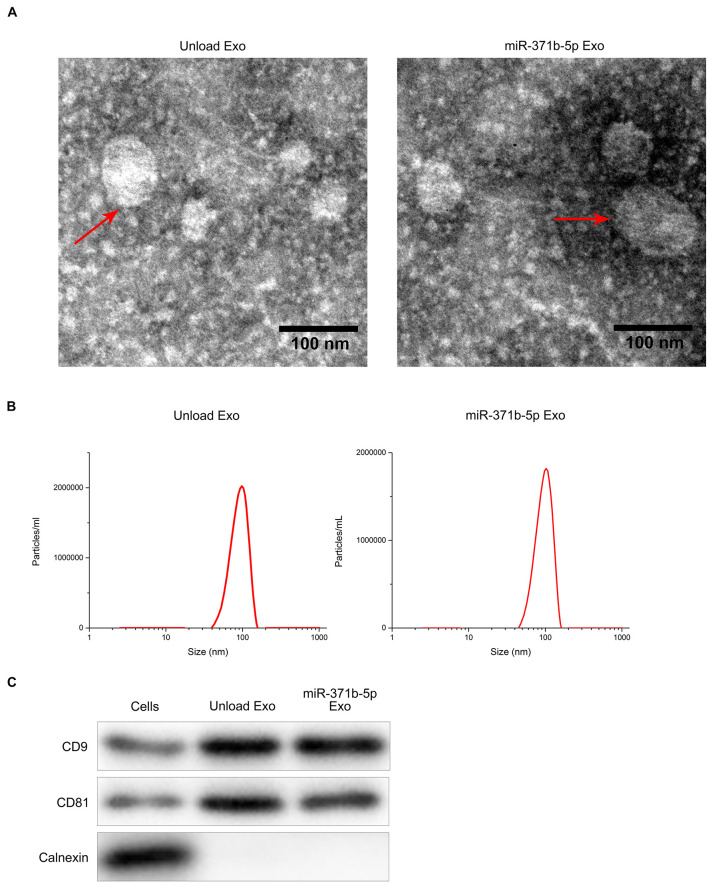
Isolation and identification of engineered exosomes. **(A)** TEM micrographs of engineered exosomes before and after loading with miR-371b-5p. **(B)** DLS assay analyzed particle size of engineered exosomes. **(C)** Western blotting assessed the levels of CD9, CD81, and Calnexin proteins that are the markers of endoplasmic reticulum.

Transmission electron microscopy (TEM) analysis showed the disk-like appearance of the engineered exosomes with the average size between 30 and 150 nm (Figure 1A). Subsequently, the purified exosomes were characterized by dynamic light scattering (DLS). DLS analysis showed that the average size of the engineered exosomes was 103 nm (Figure 1B), indicating that the secreted exosomes were successfully isolated. Furthermore, Western blot analysis showed that CD81, CD9, and other important extracellular markers were expressed in the exosomes (*P* < 0.05; Figure 1C). These findings revealed that the engineered exosomes were successfully isolated.

### Uptake of Engineered Exosomes by Tumor Cells

**FIGURE 2:**
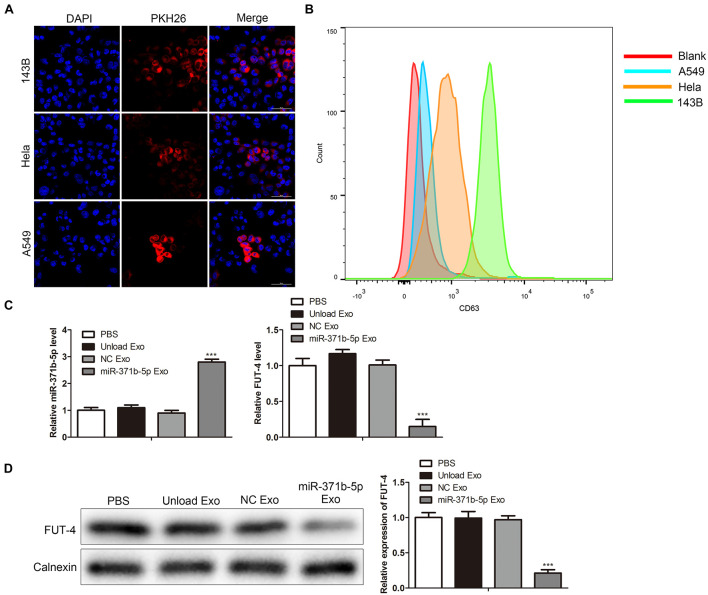
Engineered exosomes internalized by tumor cells. **(A)** Cellular uptake of PKH26-labeled engineered exosomes by 143B, Hela, and A549 cells. **(B)** FCM assay performed to evaluate the ability of tumor cells to uptake engineered exosomes. 143B cells incubated for 6 h with each of the following: PBS, unload exosomes (unload Exo), NC exosomes (NC Exo), and miR-317b-5b-loaded engineered exosomes (miR-317b-5b Exo). **(C)** RT-qPCR assessed the levels of miR-317b-5b and FUT4 in 143B cells incubated with miR-317b-5b-loaded engineered exosomes. **(D)** Western blot measured the level of FUT4 protein in 143B cells incubated with miR-317b-5b-loaded engineered exosomes. ***P < 0.001 vs. NC Exo. Error bars represented SD. Data represented three independent experiments.

Compared with other vectors, one of the unique characteristics of exosomes is that they are simply incorporated into the target cells by co-culture and penetrate the cells by endocytosis or membrane fusion (Liang et al., 2018). To investigate the specificity and efficiency of uptake of engineered exosomes by tumor cells, human osteosarcoma cell 143B, Hela cell and human lung cancer cell A549, were treated with 200 μg/mL engineered exosomes at 37°C for 24 h. The engineered exosomes were labeled with fluorescent probe PKH26. The fluorescence intensity of PKH26-labeled exosomes in tumor cells was monitored by confocal laser scanning microscope (CLSM). CLSM showed that the engineered exosomes labeled with PKH26 successfully entered 143B, Hela, and A549 cells following 24 h of coculture (Figure 2A). Flow cytometry (FCM) analysis showed that engineered exosomes marked by CD63 were taken into tumor cells, including 143B, Hela, and A549 cells (*P* < 0.05; Figure 2B). The uptake rate of PKH-67-labeled engineered exosomes was higher in 143B cells than in other cells. Thus, 143B cells were selected for subsequent functional experiments. To explore the expression of miR-317b-5b in tumor cells, miR-317b-5b level in 143B cells incubated with miR-317b-5b-engineered exosomes was detected by RT-qPCR and Western blot. The results showed that compared with the control, miR-317b-5b level was significantly up-regulated after miR-317b-5b-loaded engineered exosomes were incubated with 143B cells. It has been reported that, FUT-4 is a target of miR-371b-5p (Li et al., 2020). In order to prove the functionality of the engineered exosomes rich in miR-371b-5p FUT-4 expression level was detected. As shown in Figures 2C,D, FUT4 level in 143B cells incubated with miR-317b-5b-loaded engineered exosomes was lower than that in the control group. These findings indicated that miR-317b-5b-loaded engineered exosomes could be internalized by 143B, HeLa, and A549 cells. In the 143B cells co-incubated with miR-317b-5b-loaded engineered exosomes, miR-317b-5b level was high.

### The Effects of miR-371b-5p Delivered by Engineered Exosomes on Tumor Cell Proliferation and Apoptosis

**FIGURE 3:**
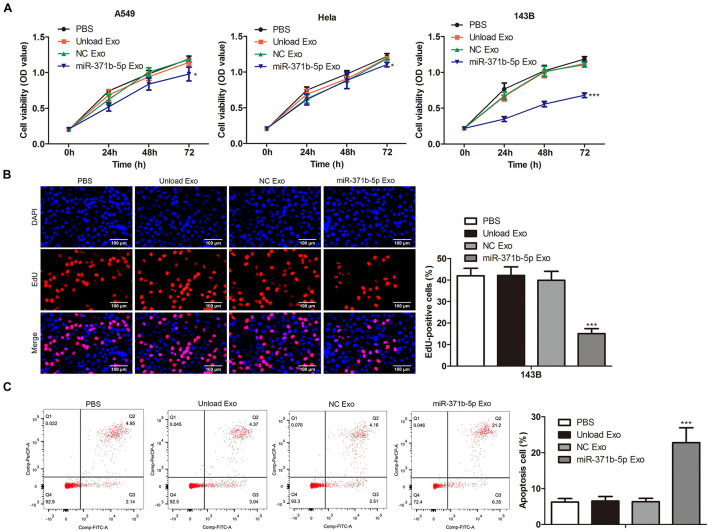
The effects of miR-371b-5p delivered by engineered exosomes on tumor cell proliferation and apoptosis. 143B, Hela, and A549 cells incubated with each of the following for 6 h: PBS, Unload Exo, NC Exo, and miR-317b-5b Exo. **(A)** CCK-8 assessed cell viability. **(B)** EdU assay on cell proliferation. **(C)** FCM evaluated cell apoptosis. *P < 0.05, ***P < 0.001 vs. NC Exo. Error bars represented SD. Data represented three independent experiments.

To further investigate the effects of miR-317b-5b-loaded engineered exosomes on the proliferation and apoptosis of 143B, Hela, and A549 cells, CCK-8 assay was performed on these cells following the incubation with miR-317b-5b-loaded engineered exosomes over 4 days. Findings showed that cell viability of 143B, HeLa, and A549 cells treated with miR-317b-5b-loaded engineered exosomes was significantly lower than that of untreated cells (*P* < 0.05; Figure 3A). The reduction of cell viability in 143B cells was more significant than the other cells. Thus, 143B cells were selected for subsequent functional experiments. EdU assay was carried out to assess the cell proliferation of 143B cells following incubation with miR-317b-5b-loaded engineered exosomes over 4 days. It was found that the rate of cell proliferation was significantly down-regulated in miR-317b-5b-loaded engineered exosomes-treated 143B cells compared to untreated cells (*P* < 0.05; Figure 3B). On the contrary, FCM analysis indicated that the rate of apoptosis was significantly up-regulated in miR-317b-5b-loaded engineered exosomes-treated 143B cells compared with the control (*P* < 0.05; Figure 3C). These findings indicated that the internalization of miR-317b-5b is accompanied by specific changes in cell biological behaviors including cell viability, proliferation, and apoptosis in 143B, HeLa, and A549 cells.

### The Effects of miR-371b-5p Delivered by Engineered Exosomes on Tumor Cell Invasion and Migration

**FIGURE 4:**
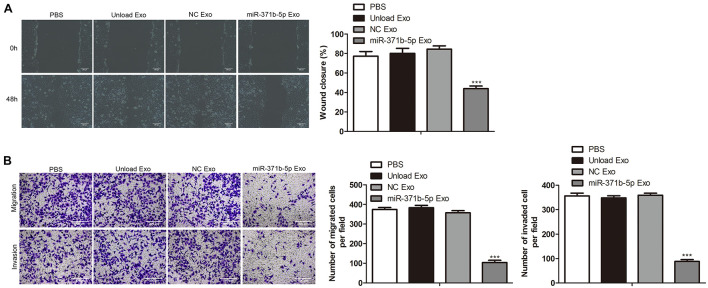
The effects of miR-371b-5p delivered by engineered exosomes on tumor cell invasion and migration. 143B cells were incubated for 6 h with each of the following: PBS, Unload Exo, NC Exo, and miR-317b-5b Exo. **(A)** Wound-healing assay assessed the effect of miR-317b-5b-loaded engineered exosomes on 143B cells migration. **(B)** Transwell assay on the effect of miR-317b-5b-loaded engineered exosomes on 143B cells migration and invasion. ***P < 0.001 vs. NC Exo. Error bars represented SD. Data represented three independent experiments.

Next, we investigated the effects of miR-317b-5b-loaded engineered exosomes by 143B cells on cell wound-healing response, invasion, and migration. Findings showed that the wound-like gaps in 143B cells of PBS, unload Exo and NC Exo groups had almost healed completely. However, compared with the control, the wound closure of 143B cells treated with miR-317b-5b-loaded engineered exosomes was significantly delayed (*P* < 0.05; Figure 4A). Similarly, transwell analysis indicated that there were significant reduction of cell invasion and migration in miR-317b-5b-loaded engineered exosomes-treated 143B cells compared to the control cells (*P* < 0.05; Figure 4B). These results suggested that the internalization of miR-317b-5b in 143B cells resulted in changes in cell invasion and migration.

### Visualization of Fluorescently Labeled Engineered Exosomes in Tumor-Bearing Mice *in vivo*

**FIGURE 5:**
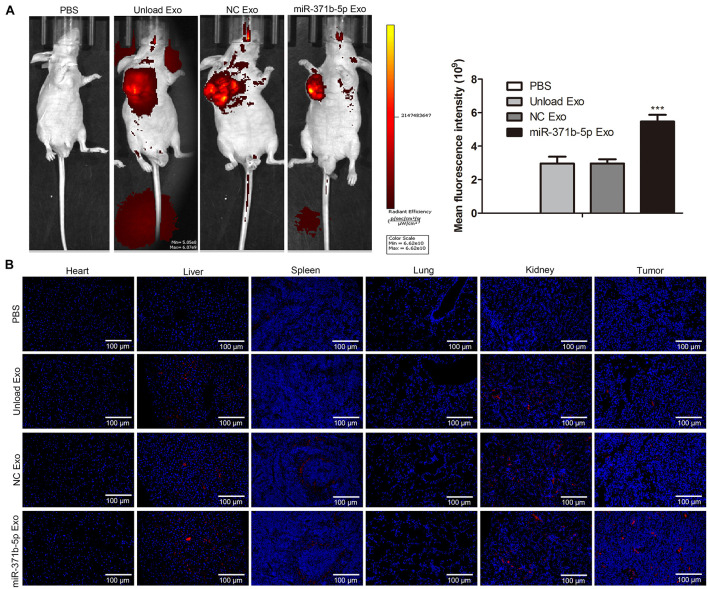
Visualization of fluorescently labeled engineered exosomes in tumor-bearing mice *in vivo*. BALB/c tumor bearing mice injected with each of the following: PBS (200 μL), unload exosomes (unload Exo; 30 μg), NC exosomes (NC Exo; 30 μg), and miR-317b-5b-loaded engineered exosomes (miR-317b-5b Exo; 30 μg). **(A)** *In vivo* miR-317b-5b-loaded engineered exosomes biodistribution in tumor-bearing mice by animal imaging. **(B)** miR-317b-5b-loaded engineered exosomes labeled with PKH26 were intravenously injected into mice bearing osteosarcoma tumors. Lung, liver, spleen, kidney, and tumor tissues were harvested after 3, 12, 24, and 48 h post injection for *ex vivo* imaging. The fluorescent signal of PKH26-labeled miR-317b-5b-loaded engineered exosomes detected using IVIS. ***P < 0.001 vs. NC Exo. Error bars represented SD. Data represented three independent experiments.

Furthermore, we evaluated the biodistribution and tumor penetration of miR-317b-5b-loaded engineered exosomes in mice with osteosarcoma. To observe the presence of miR-317b-5b engineered exosomes in tumors and tissues such as heart, liver, spleen, lung, and kidney, 10^6^ 143B cells were injected into female BALB/c mice aged 6 to 8 weeks. Mice with osteosarcoma were monitored daily. When the tumor volume reached 400mm^3^, the tumor-bearing mice were intravenously injected with 30 μg engineered exosome or 200 μL PBS containing 5% glucose as the negative control. Fluorescence intensity of engineered exosomes labeled with PKH26 was monitored from 3 to 48 h after injection. Findings indicated that compared with the control, PKH26-labeled and miR-317b-5b-loaded engineered exosomes were easily observed in different tissues and tumor sites of tumor bearing mice (Figure 5A). Subsequently, all mice, including control mice, were sacrificed at 0, 3, 12, 24, and 48 h after intravenous administration of labeled engineered exosomes or PBS, and fluorescence signals were immediately observed from fresh dissected tissues using the Interactive Video Information System (IVIS). Results showed that the fluorescent signal from PKH26-labeled and miR-317b-5b-loaded engineered exosomes were easily observed in tumor tissues and most tissues, including liver, spleen, and kidney. The fluorescent signal from PKH26-labeled and miR-317b-5b-loaded engineered exosomes in tumor tissues was the strongest (*P* < 0.05; Figure 5B). These results demonstrated that miR-317b-5b-loaded engineered exosomes were preferentially accumulated in tumor tissues.

### Anti-tumor Efficiency of Engineered Exosomes *in vivo*

**FIGURE 6:**
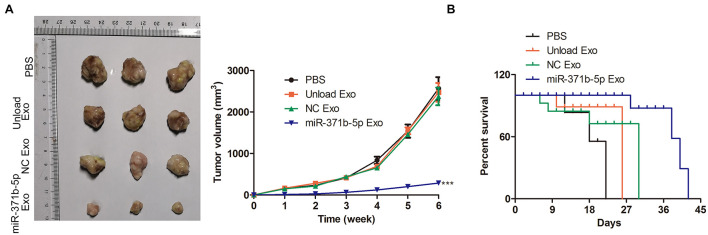
Anti-tumor efficiency of engineered exosomes *in vivo*. BALB/c tumor bearing mice were injected with each of the following: PBS (200 μL), unload exosomes (unload Exo; 30 μg), NC exosomes (NC Exo; 30 μg), and miR-317b-5b-loaded engineered exosomes (miR-317b-5b Exo; 30 μg). **(A)** Average osteosarcoma tumor volume, 16 days following infection. **(B)** Survival analyses of osteosarcoma tumor-bearing mice, 60 days following infection. ***P < 0.001 vs. NC Exo. Error bars represented SD. Data represented three independent experiments.

The antitumor effect of miR-317b-5b delivered by engineered exosomes was evaluated in the osteosarcoma mouse model. Tumor growth analysis of osteosarcoma showed that miR-317b-5b-loaded engineered exosomes inhibited tumor growth. The unload Exo and NC Exo had the same effect on tumor growth rate. Compared with PBS treated mice, tumor volume was significantly reduced in miR-317b-5b-loaded engineered exosomes-treated mice (*P* < 0.05; Figure 6A). Furthermore, survival analysis showed that the mean survival time of osteosarcoma-bearing mice treated with miR-317b-5b-loaded engineered exosomes was significantly higher than that of mice treated with PBS or Exo-unloaded and NC Exo (*P* < 0.05; Figure 6B). These findings revealed that miR-317b-5b-loaded engineered exosomes had the anti-tumor efficiency *in vivo*.

### Quantification of miR-317b-5b *in vivo*

**FIGURE 7:**
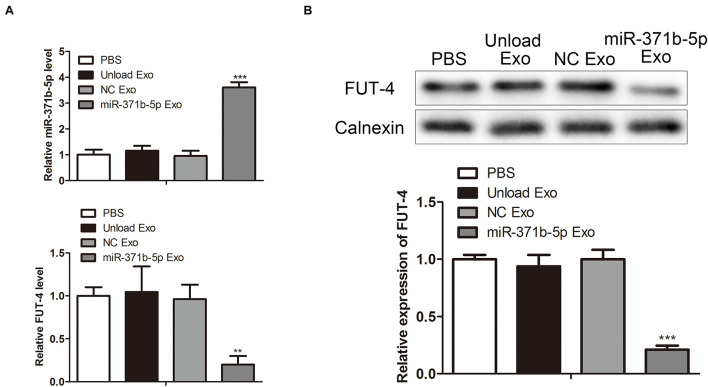
Quantification of exosome-loaded miR-317b-5b loading *in vivo*. BALB/c tumor bearing mice were injected with each of the following: PBS (200 μL), unload exosomes (unload Exo; 30 μg), NC exosomes (NC Exo; 30 μg), and miR-317b-5b-loaded engineered exosomes (miR-317b-5b Exo; 30 μg). **(A)** RT-qPCR assessed the levels of miR-317b-5b and FUT4 in tumor tissues of osteosarcoma tumor-bearing mice infected with miR-317b-5b-loaded engineered exosomes. **(B)** Western blot measured the level of FUT4 protein in tumor tissues of osteosarcoma tumor-bearing mice infected with miR-317b-5b-loaded engineered exosomes. **P < 0.01, ***P < 0.001 vs. NC Exo. Error bars represented SD. Data represented three independent experiments.

To explore the levels of miR-317b-5p in osteosarcoma tumor-bearing mice following injection with miR-317b-5b-loaded engineered exosomes, RT-qPCR and Western blot were performed to quantify the levels of miR-317b-5b and FUT4 in tumor tissues. The results showed that miR-317b-5b expression was significantly up-regulated in tumor tissues that had been injected with miR-317b-5b-loaded engineered exosomes compared with the control. On the contrary, FUT4 expression in tumor tissues that had been infected with miR-317b-5b-loaded engineered exosomes was lower than that in control group (*P* < 0.05; Figures 7A,B). These findings revealed that miR-317b-5b was overexpressed in tumor tissues of osteosarcoma tumor-bearing mice injected with miR-317b-5b-loaded engineered exosomes.

## Discussion

Exosomes are natural vehicles for the exchange of macromolecular goods and information among cells in the human body (Tkach and Théry, 2016). Proteins on exosomes may bind to target receptors on target cells, where they are internalized through receptor-mediated endocytosis (Malhotra et al., 2016). Compared with artificial nanoparticles, exosomes circulate in the body for much longer time, increasing the chance of encountering distant target cells. These favorable properties promote researchers to explore engineering exosomes to deliver exogenous nucleic acids and therapeutic drugs to diseased cells, particularly tumor cells (Tian Y. et al., 2014; Choi et al., 2016). In this study, we designed exosomes as *in vivo* vectors for tumor-targeted delivery and uptake of miRNAs therapeutic agents to tumor cells. Findings showed that miR-317b-5p was efficiently delivered to target tumor cells, leading to specific changes in tumor cell bioactivities. We further developed a simple method to design human cell-derived exosomes to deliver specific and functional therapeutic miRNA to tumor cells.

Exosomes have been recognized as carriers for a variety of nucleic acid therapeutic drugs, including siRNA, plasmid DNA, miRNA mimics, miRNA inhibitors, and mRNAs (Tian T. et al., 2014). In this study, cargo RNA was endogenously loaded into exosomes. After the RNA vector was loaded into the exosomes, the miR-317b-5p precursor sequence was modified. The introduction of TAR RNA/TAT peptide interaction between the LAMP2A fusion protein and the modified pre-miR-317b-5p loop significantly improved the delivery of pre-miR-317b-5p. Namely, we successfully constructed miR-317b-5b-loaded engineered exosomes.

It was reported that engineered exosomes delivering miRNAs play an important role in cancer diagnosis and treatment (Malla et al., 2017; Nie et al., 2020). Our findings showed that miR-317b-5b-loaded engineered exosomes were internalized by tumor cells, including A549, Hela, and 143B cells. The internalization of miR-317b-5b in tumor cells was accompanied by specific changes in cell viability, proliferation, apoptosis, migration, and invasion. Furthermore, the antitumor effect of miR-317b-5b-loaded engineered exosomes was evaluated in osteosarcoma bearing mice. According to the *in vivo* antitumor study, the lowest tumor volume and the longest survival time were observed in mice treated with miR-317b-5b-loaded engineered exosomes, which showed significant antitumor efficiency compared with the unloaded exosomes and NC exosomes. In addition, *in vivo* studies using tumor bearing mice showed that following iv injection, miR-317b-5b-loaded engineered exosomes mediated the effective delivery of miR-317b-5b to tumor tissues.

Although our research has shown that miR-317b-5b-loaded engineered exosomes is a promising treatment. However, it is important to emphasize that the clinical therapeutic potential of engineered exosomes is limited by the difficulty of their manufacture and the need for a comprehensive molecular, and functional characterization of each formulation prior to treatment, both of them are expensive and labor-intensive.

In summary, our results suggest that miR-317b-5b-loaded engineered exosomes can be used as viable nanocarriers to deliver drug molecules such as miR-317b-5b both *in vitro* and *in vivo* and exert their anti-tumor effects. The modified exosomes will eventually be made into injectable forms as therapeutic drugs, including miRNAs and protein-based therapeutic agents in the future.

## Data Availability Statement

The original contributions presented in the study are included in the article/Supplementary Material, further inquiries can be directed to the corresponding authors.

## Ethics Statement

The animal study was reviewed and approved by the Animal Ethics Committees of Nantong University.

## Author Contributions

XL and WZ designed the study. QX and YY performed the major work about mice, cell, molecular, and biochemistry experiments. LY, XY, ZS, and JL performed the molecular and biochemistry experiments. JX, XL, and WZ collected and analyzed the data. XL and WZ prepared the draft. All authors reviewed and agreed the final version of the manuscript.

## Conflict of Interest

The authors declare that the research was conducted in the absence of any commercial or financial relationships that could be construed as a potential conflict of interest.

## Publisher’s Note

All claims expressed in this article are solely those of the authors and do not necessarily represent those of their affiliated organizations, or those of the publisher, the editors and the reviewers. Any product that may be evaluated in this article, or claim that may be made by its manufacturer, is not guaranteed or endorsed by the publisher.
