# Supplementary figures and images for: miR-371b-5p-Engineered Exosomes Enhances Tumor Inhibitory Effect
Source: Front Cell Dev Biol. 2021 Oct 4;9:750171. doi: 10.3389/fcell.2021.750171 (PMC8520920; doi:10.3389/fcell.2021.750171)

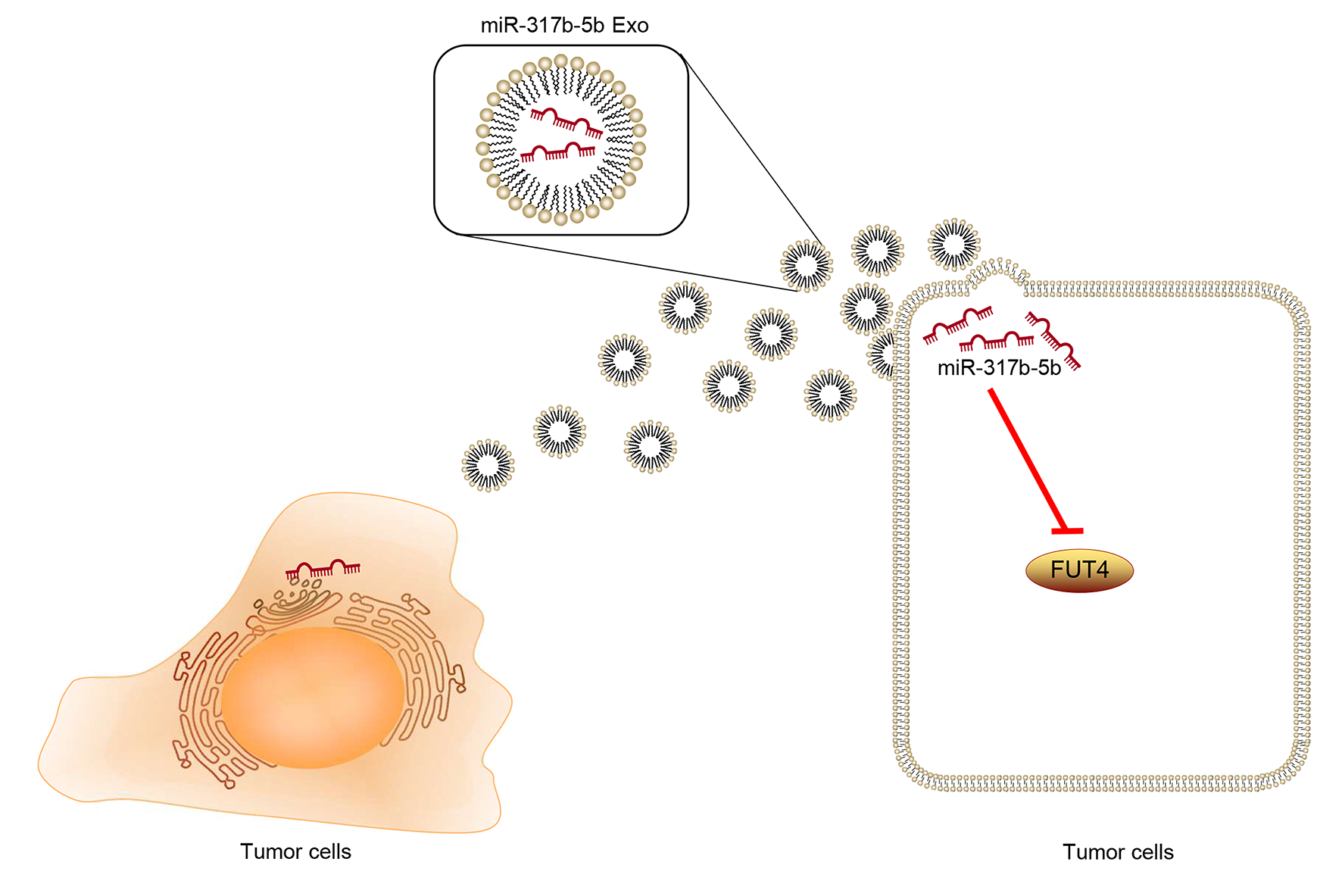

Supplement: Supplementary file 1 [file Image_1.TIF]
